# A Robust Range Grating Lobe Suppression Method Based on Image Contrast for Stepped-Frequency SAR

**DOI:** 10.3390/s16122066

**Published:** 2016-12-06

**Authors:** Wen-Bin Gao, Teng Long, Ze-Gang Ding, Yi-Rong Wu

**Affiliations:** 1 Beijing Key Laboratory of Embedded Real-Time Information Processing Technology, School of Information and Electronics, Beijing Institute of Technology, Beijing 100081, China; wbingao@gmail.com (W.-B.G.); longteng@bit.edu.cn (T.L.); 2 National Key Laboratory of Microwave Imaging Technology, Institute of Electronics, Chinese Academy of Sciences, Beijing 100080, China; wyr@mail.ie.ac.cn

**Keywords:** contrast-based, grating lobe suppression (GLS), magnitude error and phase error (MEPE), stepped-frequency, synthetic aperture radar (SAR)

## Abstract

The magnitude error and phase error (MEPE) in the transfer function of a stepped-frequency synthetic aperture radar (SAR) system results in a periodic MEPE in the synthesized wideband waveform (SWW), which induces the grating lobes in the high-resolution range profile (HRRP). In this paper, a robust data-driven grating lobe suppression (GLS) method is proposed. Based on a contrast-based error estimation method and the grating lobes of the brightest scatterers in the SAR image, the periodic MEPE can be robustly estimated using the proposed method. By compensating the estimated periodic MEPE, the range grating lobes can be suppressed to the background level of the SAR image. Simulation results and real data processing have demonstrated the superiority of the proposed method.

## 1. Introduction

By transmitting groups of narrowband linear frequency-modulated (LFM) sub-pulses and achieving synthesized wideband waveforms (SWWs) in post-processing, the stepped-frequency technique significantly reduces the requirement for an A/D converter and is widely used in improving range resolution in synthetic aperture radar (SAR) [1,2,3,4,5,6].

Periodic magnitude error and phase error (MEPE) arises in the SWW when there is a MEPE in the transfer function of a stepped-frequency SAR system. According to the paired-echo theory, periodic MEPE in the frequency domain induces pairs of grating lobes in the time domain [7]. Therefore, the grating lobes of a stepped-frequency SAR system are attributed to the periodic MEPE in the SWW. By estimating and compensating for the periodic MEPE in the SWW, the grating lobes can be suppressed remarkably. For a stepped-frequency mini-SAR system, the system volume is strictly limited and an internal calibration is not available, which requires the suppression of the range grating lobes based on raw data or a SAR image.

Several types of grating lobe suppression (GLS) methods have been proposed to account for the range grating lobes. One of the methods suppresses the range grating lobes to the desired level by restricting the relationships between the frequency step, subpulse bandwidth and subpulse-duration, which is presented in [8,9,10]. Another method sets the subpulse center frequencies at unequal spaces to eliminate the periodicity of the waveform, which disperses the grating lobes and thereby reduces the amplitudes of the grating lobes [11,12,13]. Both methods suppress the range grating lobe through the design of system parameters. The former method becomes ineffective if the transfer function of the SAR system is non-ideal, and the latter one is very complex and difficult to implement. The last method, named the grating lobe’s peak point method (GLPP), is a data-driven GLS method. It estimates the periodic MEPE based on the amplitudes and phases of a strong scatterer and its grating lobes in the SAR image [14]. However, this method requires that the periodic phase error should be smaller than 0.5 rad; otherwise the estimation accuracy of the periodic MEPE cannot be guaranteed.

Therefore, a robust data-driven GLS method is proposed in this paper, where a contrast-based autofocus algorithm is utilized. The contrast-based autofocus methods have shown to be more robust than standard autofocus methods, under stressing conditions, (such as low image contrast, a significant amount of noise, and substantial phase errors) [15,16,17]. However, the existing contrast-based autofocus methods are all considered for the phase error, with the amplitude error remaining as a problem. Therefore, a novel contrast-based amplitude error estimation method is proposed in this paper to guarantee the estimation accuracy of the periodic MEPE.

This paper is structured as follows: in Section 2, the GLS problem is defined as a contrast-based autofocus problem; a robust GLS method based on a contrast-based autofocus algorithm is proposed in Section 3; computer simulations are discussed in Section 4, and an experiment based on real data is reported in Section 5; and, finally, the conclusion is presented in Section 6.

## 2. GLS Problem Definition

The periodic MEPE $H\left(f_{r}\right)$ in the SWW can be written as [14]:

(1) $$H\left(f_{r}\right)=\sum_{n=0}^{N-1}\{rect\left(\left(f_{r}-f_{n}\right)/\Delta f\right)\cdot A\left(f_{r}-f_{n}\right)\cdot \exp\left[j\theta \left(f_{r}-f_{n}\right)\right]\},$$

where $f_{r}$ represents the range frequency, *N* represents the number of subpulses in a pulse group, $f_{n}$ represents the frequency shift of the *n*-th subpulse during spectrum reconstruction, $A\left(f_{r}\right)$ and $\theta \left(f_{r}\right)$ represent the magnitude error and phase error of the subpulse, respectively.

The SWW $S_{comb}\left(f_{r}\right)$ is written as:

(2) $$S_{comb}\left(f_{r}\right)=S_{ideal}\left(f_{r}\right)\cdot H\left(f_{r}\right),$$

where $S_{ideal}\left(f_{r}\right)$ represents the ideal synthesized wideband spectrum without MEPE.

The high-resolution range profile (HRRP) $s_{comb}(t)$ of the stepped-frequency SAR is the inverse Fourier transform of the SWW $S_{comb}\left(f_{r}\right)$:

(3) $$s_{comb}\left(t\right)=\int_{-\infty }^{+\infty }S_{comb}\left(f_{r}\right)\cdot \exp\left(j2\pi f_{r}t\right)df_{r},$$

and $s_{comb}(t)$ is written as:

(4) $$\begin{array}{c} s_{comb}(t)=s\left(t\right)+\sum_{n=1}^{\infty }\{s\left(t+nc_{1}\right)\cdot \left[\frac{b_{n}}{2}\cdot \exp\left(j\frac{\pi }{2}+j\Phi_{bn}\right)+\frac{a_{n}}{2a_{0}}\cdot \exp\left(j\Phi_{an}\right)\right]\\ \;+s\left(t-nc_{1}\right)\cdot \left[\frac{b_{n}}{2}\cdot \exp\left(j\frac{\pi }{2}-j\Phi_{bn}\right)+\frac{a_{n}}{2a_{0}}\cdot \exp\left(-j\Phi_{an}\right)\right]\} \end{array},$$

where s(t) is the inverse Fourier transform of $S_{ideal}\left(f_{r}\right)$, and represents the main lobe; $a_{0}$, $a_{n}$, $b_{n}$, $c_{1}$, $\Phi_{an}$, and $\Phi_{bn}$ are some coefficients determined by $H\left(f_{r}\right)$, which determines the positions, amplitudes and phases of the grating lobes.

The discrete form of Equation (3) is written as:

(5) $$s_{comb}\left(m\right)=\frac{1}{N_{r}}\sum_{m=0}^{N_{r}-1}S_{comb}\left(p\right)\cdot \exp\left(j\frac{2\pi }{N_{r}}mp\right),$$

where $N_{r}$ represents the range sample number of HRRP, *m* and *p* refer to the range time and range frequency, respectively.

The problem of range GLS can be formulated as:

(6) $$s_{GLS}\left(m\right)=\frac{1}{N_{r}}\sum_{m=0}^{N_{r}-1}S_{comb}\left(p\right)\cdot H_{GLS}\left(p\right)\cdot \exp\left(j\frac{2\pi }{N_{r}}mp\right),$$

where $H_{GLS}\left(p\right)$ is utilized to compensate for the periodic MEPE.

To achieve a robust estimation of $H_{GLS}\left(p\right)$ under low image signal-to-noise ratio (SNR) and large background noise conditions, a contrast-based error estimation method is utilized in the proposed GLS method, which searches for $H_{GLS}\left(p\right)$ by maximizing the image contrast *C*. It is found that the contrast-based calibration algorithm converges faster than the entropy-based calibration algorithm based on our experience processing real data. Furthermore, it has been proved that maximizing the expected value of the 4-norm contrast metric is equivalent to phase-calibrating the image [18]. Therefore, the intensity-based contrast function described in [19] is used in this paper. Assuming that the stepped-frequency SAR image $s_{GLS}\left(m,\;k\right)$ consists of $N_{a}$ azimuth samples, the image contrast *C* is written as:

(7) $$C=\frac{\sqrt{N_{a}N_{r}\sum_{k=0}^{N_{a}-1}\sum_{m=0}^{N_{r}-1}{\left|s_{GLS}\left(m,k\right)\right|}^{4}-{\left[\sum_{k=0}^{N_{a}-1}\sum_{m=0}^{N_{r}-1}{\left|s_{GLS}\left(m,k\right)\right|}^{2}\right]}^{2}}}{\sum_{k=0}^{N_{a}-1}\sum_{m=0}^{N_{r}-1}{\left|s_{GLS}\left(m,k\right)\right|}^{2}},$$

where *k* refers to the azimuth time.

## 3. The Proposed GLS Method

$H_{GLS}\left(p\right)$ can be divided into two parts: the amplitude part $H_{AMP}\left(p\right)$ and the phase part $\exp\left(j\cdot H_{PHA}\left(p\right)\right)$. A direct search for both of the two parts is quite time-consuming since the image contrast defined as Equation (7) is quite complex. A computational efficient method is to estimate the two parts separately, and $H_{GLS}\left(p\right)$ can be achieved by combining the two parts.

As we know, autofocusing synthetic aperture imagery by maximizing a statistical quality metric, such as contrast or entropy, is a well-documented approach in the synthetic aperture radar literature [18,19,20,21,22,23,24,25,26]. During these publications, the optimization approach adopted in [23,24,26] is the monotonic iterative technique, which is computationally efficient and achieves a quick convergence. Therefore, the optimization approach of monotonic iterative technique is utilized in this paper.

### 3.1. Contrast-Based Phase Error Estimation

When merely $H_{PHA}\left(p\right)$ is to be estimated, the problem of range GLS is formulated as:

(8) $$s_{GLS}\left(m,\;k\right)=\frac{1}{N_{r}}\sum_{p=0}^{N_{r}-1}S_{comb}\left(p,\;k\right)\cdot \exp\left(j\cdot H_{PHA}\left(p\right)\right)\cdot \exp\left(j\frac{2\pi }{N_{r}}mp\right).$$

According to the Parseval theorem, $\sum_{k=0}^{N_{a}-1}\sum_{m=0}^{N_{r}-1}{\left|s_{GLS}\left(m,k\right)\right|}^{2}=\sum_{k=0}^{N_{a}-1}\sum_{p=0}^{N_{r}-1}{\left|S_{comb}\left(p,\;k\right)\right|}^{2}=Const$ is a constant value for a given image. Thus, the gradient of the image contrast with respect to $H_{PHA}\left(p\right)$ is written as:

(9) $$\frac{\partial C}{\partial H_{PHA}\left(p\right)}=\frac{N_{a}N_{r}/\left(2Const\right)}{\sqrt{N_{a}N_{r}\sum_{k=0}^{N_{a}-1}\sum_{m=0}^{N_{r}-1}{\left|s_{GLS}\left(m,k\right)\right|}^{4}-Const^{2}}}\cdot \frac{\partial \sum_{k=0}^{N_{a}-1}\sum_{m=0}^{N_{r}-1}{\left|s_{GLS}\left(m,k\right)\right|}^{4}}{\partial H_{PHA}\left(p\right)}.$$

The image contrast can be maximized in an iterative way [23,24,26], in which the temporary estimation of $H_{PHA}\left(p\right)$ in each iteration is achieved by letting $\partial C/\partial H_{PHA}\left(p\right)=0$, and the temporary estimation of $H_{PHA}\left(p\right)$ is written as:

(10) $$\exp\left(j\cdot {\hat{H}}_{PHA}\left(p\right)\right)=\frac{O^{*}\left(m\right)}{\left|O\left(m\right)\right|}$$

where

(11) $$O\left(m\right)=\frac{1}{N_{r}}\sum_{k=0}^{N_{a}-1}S_{comb}\left(p,\;k\right)\cdot conj\left\{DFT_{m}\left[{\left|s_{GLS}\left(m,\;k\right)\right|}^{2}s_{GLS}\left(m,\;k\right)\right]\right\},$$

and conj refers to the complex conjuction, $DFT_{m}$ refers to the discrete Fourier transform, with the subscript denoting the dimension in which the transform is applied.

By compensating $\exp\left(j\cdot {\hat{H}}_{PHA}\left(p\right)\right)$ into the SWW, the image contrast can be improved. When the contrast difference between the two images of two adjacent iterations is smaller than a set threshold, the iterative estimating process can be stopped, and the estimated ${\hat{H}}_{PHA}\left(p\right)$ of all iterations are combined to obtain the final ${\hat{H}}_{PHA}\left(p\right)$.

### 3.2. Contrast-Based Amplitude Error Estimation

When merely $H_{AMP}\left(p\right)$ is to be estimated, the problem of range GLS is formulated as:

(12) $$s_{GLS}\left(m,\;k\right)=\frac{1}{N_{r}}\sum_{m=0}^{N_{r}-1}S_{comb}\left(p,\;k\right)\cdot H_{AMP}\left(p\right)\cdot \exp\left(j\frac{2\pi }{N_{r}}mp\right).$$

Since $H_{AMP}\left(p\right)$ changes the amplitude of the image, $\sum_{k=0}^{N_{a}-1}\sum_{m=0}^{N_{r}-1}{\left|s_{GLS}\left(m,k\right)\right|}^{2}$ is never a constant. Thus, the gradient of the image contrast with respect to $H_{AMP}\left(p\right)$ is written as:

(13) $$\frac{\partial C}{\partial H_{AMP}\left(p\right)}=\frac{N_{a}N_{r}}{2\sqrt{N_{a}N_{r}\frac{A}{B}-1}}\cdot \{\frac{1}{B}\cdot \frac{\partial A}{\partial H_{AMP}\left(p\right)}-\frac{A}{B^{2}}\cdot \frac{\partial B}{\partial H_{AMP}\left(p\right)}\},$$

where *A* represents the fouth power of the 4-norm of the image matrix, and *B* represents the fourth power of the 2-norm of the image matrix, $\partial A/\partial H_{AMP}\left(p\right)$ represents the gradient of *A* with respect to $H_{AMP}\left(p\right)$, $\partial B/\partial H_{AMP}\left(p\right)$ represents the gradient of *B* with respect to $H_{AMP}\left(p\right)$. *A*, *B*, $\partial A/\partial H_{AMP}\left(p\right)$, and $\partial B/\partial H_{AMP}\left(p\right)$ are expressed as:

(14) $$A=\sum_{k=0}^{N_{a}-1}\sum_{m=0}^{N_{r}-1}{\left|s_{GLS}\left(m,k\right)\right|}^{4},$$

(15) $$B={\left[\sum_{k=0}^{N_{a}-1}\sum_{m=0}^{N_{r}-1}{\left|s_{GLS}\left(m,k\right)\right|}^{2}\right]}^{2},$$

(16) ∂A∂HAMP(p)=4NrRe{∑k=0Na−1Scomb(p, k)⋅conj(DFTm[|sGLS(m,k)|2sGLS(m,k)])},

(17) ∂B∂HAMP(p)=4NrRe{∑k=0Na−1∑m=0Nr−1|sGLS(m,k)|2⋅∑k=0Na−1Scomb(p, k)⋅conj(DFTm[sGLS(m,k)])}.

Letting $\frac{\partial C}{\partial H_{AMP}\left(p\right)}=0$, an estimate of $H_{AMP}\left(p\right)$ is achieved, which is written as

(18) H^AMP(p)=Re{BA⋅∑k=0Na−1Scomb(p, k)⋅conj(DFTm[|sGLS(m,k)|2sGLS(m,k)])∑k=0Na−1|Scomb(p, k)|2}.

By estimating and compensating $H_{AMP}\left(p\right)$ iteratively, the image contrast is improved rapidly. When the contrast difference between the two images of two adjacent iterations is smaller than a set threshold, the estimating process can be stopped, and the estimated ${\hat{H}}_{AMP}\left(p\right)$ of all iterations are combined to get the final ${\hat{H}}_{AMP}\left(p\right)$.

The final estimation of $H_{GLS}\left(p\right)$ is achieved by:

(19) $${\hat{H}}_{GLS}\left(p\right)={\hat{H}}_{AMP}\left(p\right)\cdot \exp\left(j\cdot {\hat{H}}_{PHA}\left(p\right)\right).$$

Since the second accumulation part in Equation (11) and the numerator of the second term in Equation (18) can be calculated by fast Fourier transformation, the methods in searching for $H_{PHA}\left(p\right)$ and $H_{AMP}\left(p\right)$ are quite computationally efficient.

### 3.3. GLS Method Description

At first, a coarse image is achieved using conventional SAR imaging algorithms [27,28], and M brightest scatterers with their grating lobes are searched for in the coarse image.

Then, a discrete rectangular window covering the main lobe and grating lobes is adopted, which preserves the information of $H\left(f_{r}\right)$ and, meanwhile, suppresses the noise and interference from the neighboring clutter. Equation (4) shows that the information of $H\left(f_{r}\right)$ is reflected by the positions, amplitudes and phases of the grating lobes. This means that the periodic MEPE $H\left(f_{r}\right)$ is able to be recovered from the range grating lobes, which is the foundation of the proposed GLS method in this paper.

The next step is to estimate the amplitude error and phase error in an iterative way, which is described as the following steps:

Step 1: Initialize the phase error ${\hat{H}}_{PHA\_l}\left(p\right)$, where l is the iteration number.
Step 2: Use Equation (8) to compensate for the phase error.
Step 3: Calculate the contrast of image $s_{GLS\_l}$. If l is greater than 1 and $C_{GLS}\left(l\right)-C_{GLS}\left(l-1\right)$ is greater than a pre-set threshold, then go to Step 4; otherwise, stop.
Step 4: Use Equation (10) to achieve the phase error estimation ${\hat{H}}_{PHA\_l}\left(p\right)$.
Step 5: Update l with l+1, and go to Step 2.

The above steps describe the process of phase error estimation. When the phase error ${\hat{H}}_{PHA\_l}\left(p\right)$ is replaced by amplitude error ${\hat{H}}_{AMP\_l}\left(p\right)$, Equation (8) is replaced by Equation (12), and Equation (10) is replaced by Equation (18), the above steps are also suitable for the estimation of amplitude error.

**Figure 1:**
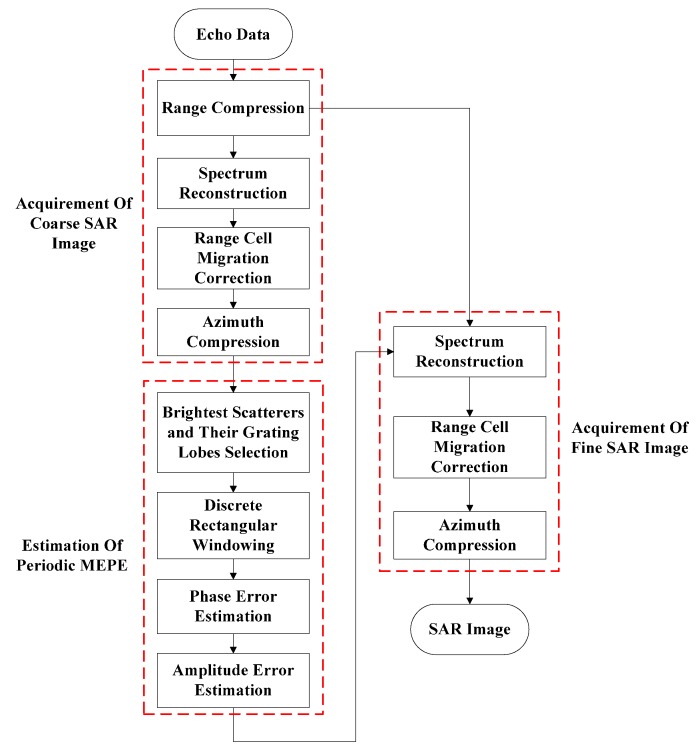
Flowchart for the developed GLS method.

Finally, the estimated MEPE are compensated during the spectrum reconstruction operation, and the fine SAR image with range grating lobes suppressed is achieved by conventional imaging algorithms. The flowchart of the proposed GLS method is shown in Figure 1.

It needs to be pointed out that the selected strong scatterers are not equivalent to point scatterers, a simple spectral density equalization of the different sub-pulses eliminates the original amplitude error, but introduces an unexpected amplitude error into the sub-pulses at the same time, which will be demonstrated by experimental data processing in Section 5. 

## 4. Computer Simulations

The validity of the proposed GLS method is verified by computer simulations in this section. The added MEPE during simulations are extracted from a real stepped-frequency SAR. In the SAR system, 24 subpulses were used in each pulse group, with a frequency step of 20 MHz. 

**Figure 2:**
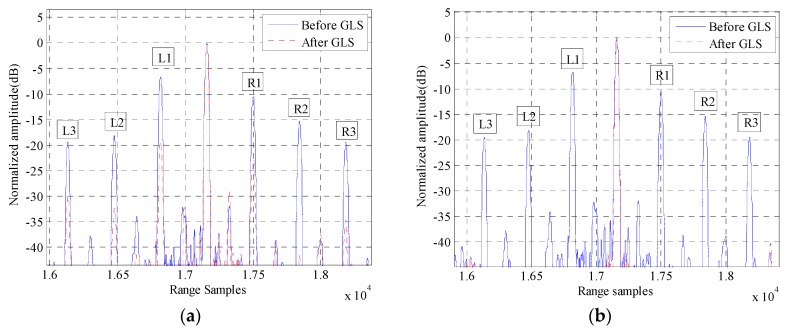
The HRRPs of a target before and after GLS: (**a**) GLS by GLPP; and (**b**) GLS by the proposed method.

**Figure 3:**
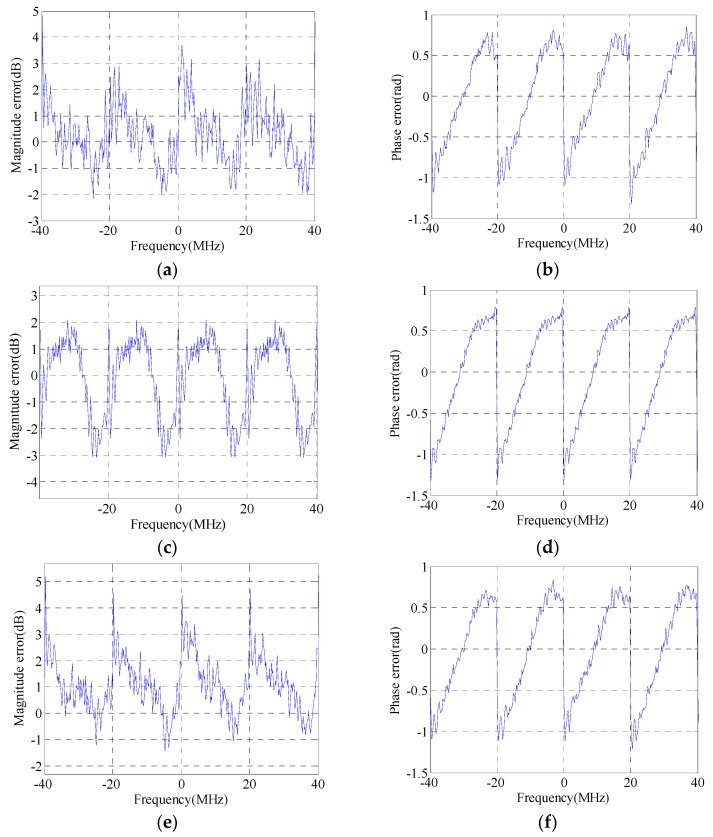
MEPE in SWW: (**a**) added magnitude error; (**b**) added phase error; (**c**) estimated magnitude error by GLPP; (**d**) estimated phase error by GLPP; (**e**) estimated magnitude error by the proposed method; and (**f**) estimated phase error by the proposed method.

The HRRPs (with a hamming window added during matched filtering) of a simulated target before and after GLS are illustrated in Figure 2. Figure 2a,b represents the GLS results of GLPP and the proposed method, respectively. The magnitudes of the grating lobes before and after GLS are listed in Table 1. The real MEPEs added into simulations are shown in Figure 3a,b, and the estimated MEPEs by GLPP and the proposed method are shown in Figure 3c–f.

It is found from Figure 2 and Figure 3 and Table 1 that the proposed GLS method is superior to GLPP, since a better GLS result is achieved by the proposed GLS method. It is also found that the magnitudes of the first pair of grating lobes after GLS by GLPP are quite large; that is because GLPP is based on the assumption that the periodic phase error is smaller than 0.5 rad [14], which is invalid in this simulation. Meanwhile, the proposed GLS method can estimate the MEPE accurately regardless of that assumption, which shows that the proposed GLS method is more robust than GLPP.

The maximum variations of the added magnitude error and phase error shown in Figure 3a,b are almost 5 dB and 2 rad, respectively, which are quite large for a real SAR system. In this extreme condition, the proposed method still works very well. Therefore, a conclusion can be drawn that the proposed GLS method can always estimate the MEPE accurately.

## 5. Experimental Data Processing

An experiment based on a real stepped-frequency SAR system was conducted to verify the validity of the proposed GLS method. The SAR system was mounted on an unmanned aerial vehicle (UAV). It was operated in the Ku-band and worked in the strip-map mode. The number of subpulses in each pulse group was 48, and the frequency step was 20 MHz. Due to the limited volume and power consumption, internal calibration is not available for this UAV SAR system. Since the intermediate-frequency amplifier of the stepped-frequency SAR system is non-ideal, an MEPE is induced in the transfer function of the system. Thus, the periodic MEPE appears in the SWW, which induces the grating lobes in the HRRP.

**Figure 4:**
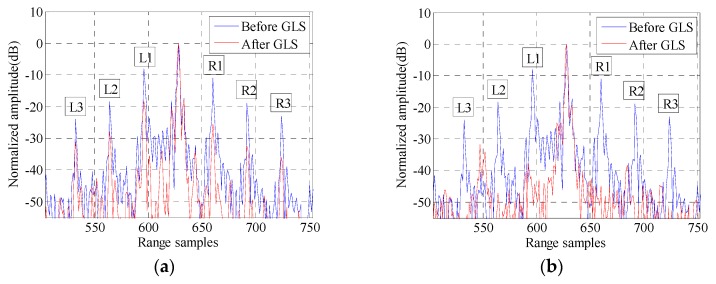
The HRRPs of target 2 before and after GLS: (**a**) GLS by GLPP; and (**b**) GLS by the proposed method.

The HRRPs of target 2 (marked yellow in the following two-dimensional SAR image) before and after GLS in real data processing are illustrated in Figure 4, where Figure 4a,b represents the GLS results of GLPP and the proposed method, respectively. The magnitudes of the grating lobes before and after GLS are listed in Table 2. Figure 4 and Table 2 show that the proposed GLS method is superior to GLPP in suppressing the range grating lobes.

**Figure 5:**
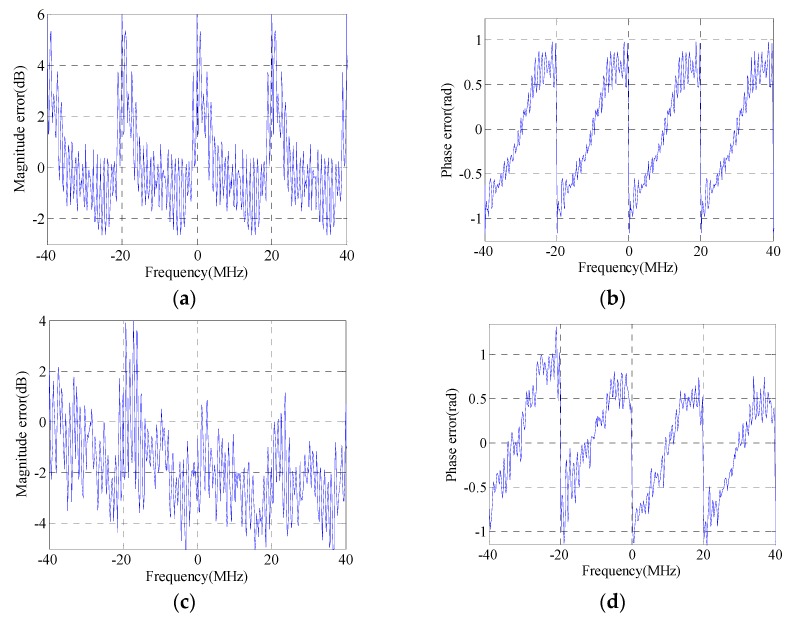
Estimated MEPE: (**a**) estimated magnitude error by GLPP; (**b**) estimated phase error by GLPP; (**c**) estimated magnitude error by the proposed method; and (**d**) estimated phase error by the proposed method.

**Figure 6:**
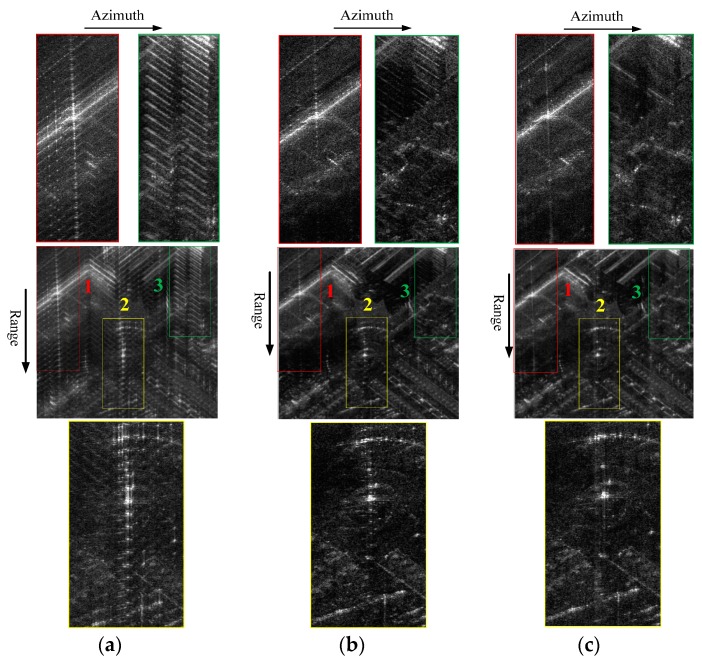
Stepped-frequency SAR image: (**a**) before GLS; (**b**) after GLS by GLPP; and (**c**) after GLS by the proposed method.

The estimated MEPEs by GLPP and the proposed method are illustrated in Figure 5. The SAR images before GLS and after GLS are given in Figure 6. By comparing Figure 6b,c, one can find that the proposed method has a higher estimation accuracy of the MEPE than GLPP. The enlarged areas in Figure 6c indicate that the range grating lobes are suppressed to the background level of the SAR image by the proposed method without influencing the azimuth focusing.

**Figure 7:**
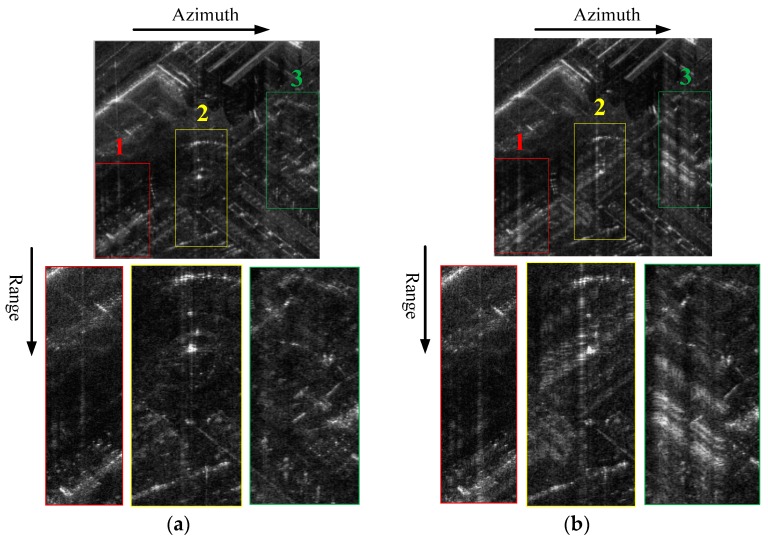
Stepped-frequency SAR image after GLS: (**a**) by the proposed method; and (**b**) by the contrast-based phase error estimation method combined with a spectral density equalization.

The SAR images after GLS are illustrated in Figure 7, where Figure 7a is the GLS result by the proposed method and Figure 7b is the GLS result by the contrast-based phase error estimation method combined with a spectral density equalization. By comparing Figure 7a and Figure 7b, one can find that the range grating lobes are both suppressed to the background level of the SAR image. However, the HRRPs of Figure 7b are obscure and the original scene is damaged. Since the strong scatterers selected in this imaging scene are not equivalent to point scatterers, an unexpected amplitude error is introduced into the echo data by the spectral density equalization operation, which damaged the observed scene.

## 6. Conclusions 

A robust data-driven range grating lobe suppression method is proposed for the stepped-frequency SAR in this paper. Based on a contrast-based error estimation method and the grating lobes of the brightest scatterers in the SAR image, the periodic MEPE in the SWW can be accurately estimated and compensated using the proposed GLS method. The simulation results and real data processing demonstrate that the proposed method is superior to GLPP in suppressing the range grating lobes, and can robustly suppress the grating lobes induced by the MEPE to the background level of the SAR image. Future work will include extending the novel contrast-based amplitude error estimation method for motion error estimation in high-resolution UAV SAR imaging, where a gimbal may be unavailable due to the strict restriction on the system weight and volume.

## Figures and Tables

**Table 1:** Magnitudes of grating lobes before and after GLS.

Different Lobes	Magnitudes of Grating Lobes before GLS in Figure 2 (dB)	Magnitudes of Grating Lobes after GLS by GLPP in Figure 2a (dB)	Magnitudes of Grating Lobes after GLS by the Proposed Method in Figure 2b (dB)
L1	−7.005	−18.58	−37.75
R1	−10.84	−22.95	−38.11
L2	−18.56	−32.80	−43.00
R2	−15.76	−35.59	−44.05
L3	−20.03	−29.84	−46.63
R3	−19.75	−32.68	−45.91

**Table 2:** Magnitudes of grating lobes before and after GLS.

Different Lobes	Magnitudes of Grating Lobes before GLS in Figure 4 (dB)	Magnitudes of Grating Lobes after GLS by GLPP in Figure 4a (dB)	Magnitudes of Grating Lobes after GLS by the Proposed Method in Figure 4b (dB)
L1	−8.09	−18.23	−37.74
R1	−10.87	−25.57	−44.86
L2	−18.26	−27.94	−47.11
R2	−18.88	−32.33	−47.99
L3	−23.95	−31.16	−53.89
R3	−23.00	−36.04	−51.20
